# Early integration of the individual student in academic activities: a novel classroom concept for graduate education in molecular biophysics and structural biology

**DOI:** 10.1186/2046-1682-7-6

**Published:** 2014-08-05

**Authors:** Sanford H Leuba, Sean M Carney, Elizabeth M Dahlburg, Rebecca J Eells, Harshad Ghodke, Naveena Yanamala, Grant Schauer, Judith Klein-Seetharaman

**Affiliations:** 1 Graduate Program in Molecular Biophysics and Structural Biology, University of Pittsburgh and Carnegie Mellon University, Pittsburgh, PA 15260, USA; 2 Department of Cell Biology, University of Pittsburgh School of Medicine, UPCI, Hillman Cancer Center, 5117 Centre Avenue, Pittsburgh, PA 15213, USA; 3 Presently at: Single Molecule Biophysics Group, Zernike Institute for Advanced Materials, University of Groningen, Groningen, The Netherlands; 4 Department of Structural Biology, University of Pittsburgh, Pittsburgh, USA; 5 Presently at: Department of Biomedicine and Systems Biology, Division of Metabolic and Vascular Health, Warwick Medical School, University of Warwick, Coventry CV4 7AL, UK

**Keywords:** Teaching, Interdisciplinary education, Molecular biophysics and structural biology, Student lectures

## Abstract

**Background:**

A key challenge in interdisciplinary research is choosing the best approach from a large number of techniques derived from different disciplines and their interfaces.

**Results:**

To address this challenge in the area of Biophysics and Structural Biology, we have designed a graduate level course to teach students insightful use of experimental biophysical approaches in relationship to addressing biological questions related to biomolecular interactions and dynamics. A weekly seminar and data and literature club are used to compliment the training in class. The course contains wet-laboratory experimental demonstration and real-data analysis as well as lectures, grant proposal preparation and assessment, and student presentation components. Active student participation is mandatory in all aspects of the class. Students prepare materials for the class receiving individual and iterative feedback from course directors and local experts generating high quality classroom presentations.

**Conclusions:**

The ultimate goal of the course is to teach students the skills needed to weigh different experimental approaches against each other in addressing a specific biological question by thinking and executing academic tasks like faculty.

## Background

Biology no longer only comprises zoology, botany, and physiology but is more and more infiltrated by many other disciplines, such as physics, computer science and engineering as attested by the coining of hundreds of sub-disciplines ranging from biochemistry, biological chemistry, bioinformatics, computational biology, biostatistics, mathematical biology, biophysics, biophysical chemistry, structural biology, biomaterials, bioengineering, bioorganic chemistry, bioinorganic chemistry biological physics, systems biology, biomedicine, immunology, cell biology, pharmacology to the recent explosion in –omics disciplines. While this has resulted in unprecedented leaps of progress due to the cross-fertilization with approaches developed originally for tasks in other disciplines, it also results in the challenge of a much larger repertoire of approaches being available to choose from to address a particular research question. Many questions related to the molecular nature of biological molecules are related to biomolecular interactions and dynamics, and addressing such questions thus both benefits and suffers from this broad spectrum of available techniques. The challenge then becomes how to teach students the skills needed to carry out research in addressing such questions. Here, we present a course designed to address this challenge. We recognize the diversity in educational backgrounds that the students are coming from, and integrate one-on-one contact with course directors and other local faculty as well as encourage an unusually high level of class participation by the students. The ultimate goal of the course is to teach students the skills needed to weigh different experimental approaches against each other in addressing a specific biological question. They should know when a specific technique is applicable and when it is not (or only in a limited fashion), know what information is obtainable from a specific technique and what is not, and how these different techniques complement each other. They should have a basic understanding of some of the major types of questions that are being addressed in modern molecular biophysics and structural biology, how the current knowledge in each field has been obtained, and what are open questions. They should be able to come up ad hoc with reasonable suggestions for how these open questions could be addressed. Here, we describe our approaches towards these goals.

## Graduate program context

**Figure 1:**
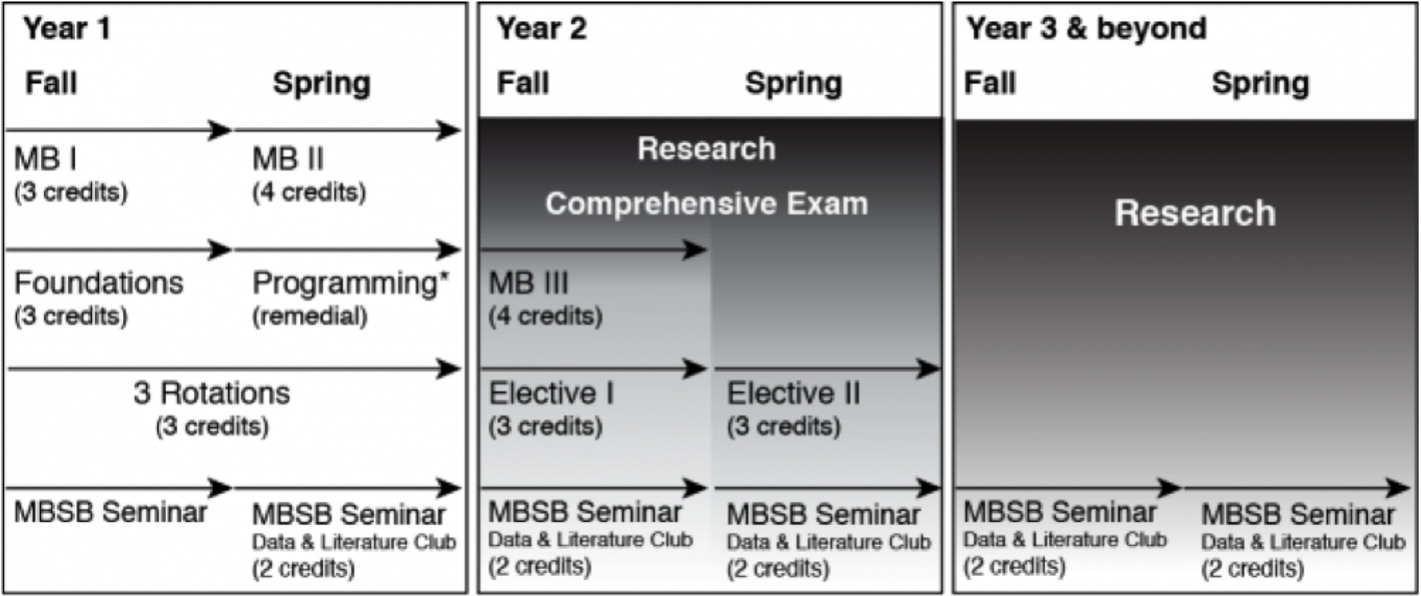
**Overview of the MBSB curriculum.** MB stands for Molecular Biophysics. For more information on classes in the program, see http://www.mbsb.pitt.edu. It is the MB II class that is described in this paper.

Students with a quantitative interest in the precise molecular nature underlying the structures and functions of biological molecules may gravitate towards graduate programs with emphasis on the areas of biophysics and structural biology such as those listed on the Biophysical Society website ( http://www.biophysics.org/). Each of these graduate programs has developed approaches to teaching their graduate students the basic knowledge that will enable their success in research and science in biophysics [1-7]. The Molecular Biophysics (MB) and Structural Biology (MBSB) Graduate Program, jointly offered by the University of Pittsburgh and Carnegie Mellon University, has developed one suitable curriculum, outlined in Figure 1. There are three core classes, referred to as MB I, MB II and MB III. The MB I class introduces first year graduate students to the most widely used approaches in biophysics and structural biology such as various spectroscopic methods and structure determination techniques such as NMR, X-ray crystallography, cryo-electron microscopy, fluorescence microscopy, and single molecule approaches. The second core class, MB II, is entitled "Biomolecular Interactions and Dynamics", and is the focus of this article. The third class, MB III, focuses on methods employed in Computational Biology [8]. First year students also take a computational programming class in case they lack prior programming experience and a course on the foundations of biomedical sciences in which the general principles of molecular and cellular biology are reviewed. Additionally, the MBSB program holds a weekly Data and Literature Club in which the students present their data as well as relevant literature for the weekly invited seminar speaker (see below). Other program requirements are attendance of advanced classes, research rotations, a comprehensive examination, followed by thesis proposal and the ensuing PhD focus.

## Methods

**Figure 2:**
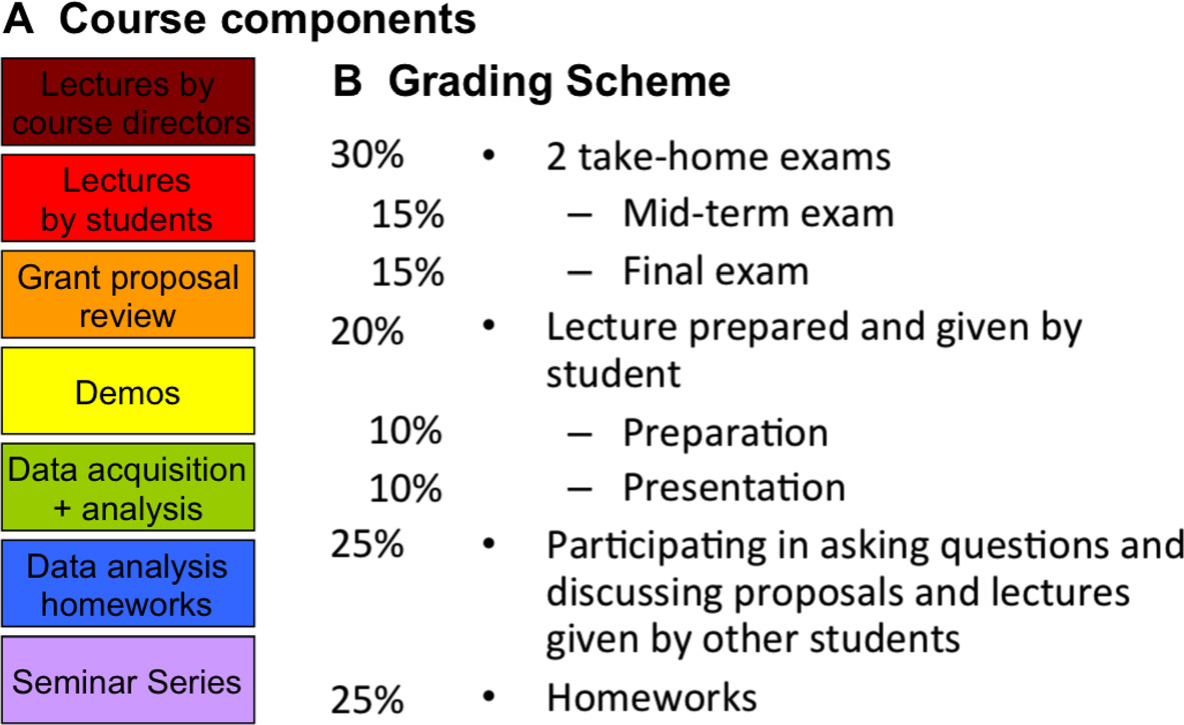
Overview of course components (A) and the grading scheme (B).

The apparent topic of the MB II class is to teach the students about the role and experimental characterization of biomolecular interactions and dynamics, building on the first course’s teaching of protein structure and the major techniques used in molecular biophysics. Because the topic is vast and the choice of systems to be discussed is endless, another and in fact primary motivation is to facilitate a transition from being a student to becoming a critical, independent thinker, necessary for each student’s progression to an independent scientist and - where desired - faculty. The major tasks that independent scientists and academic faculty in particular, engage in are performing and/or supervising experiments, writing manuscripts for submission to peer-reviewed journals, giving presentations about our research, and teaching (in addition to non-scientific tasks, such as managerial, administrative and political chores). The skill set required to perform these tasks is typically ad hoc, and we therefore have designed the MB II course to help students acquire these skills in a more streamlined process than usual. A primary element of the course is the emphasis on the individual student. For this, we have evolved a course that puts much of the control of the content covered in class into the hands of the students, with adequate supervision, guidance, and feed-back by the course directors.The course structure is outlined in Figure 2. There are some lectures by the course directors to keep a "red thread" throughout the course, as well as facilitate discussions on approaches. In particular, we give credit to the fact that there has been a transformation of the molecular bioscience field with the use of cutting-edge physics-based single molecule technologies. For example, roughly half of the topics of the Biophysical Society Annual Meeting have incorporated single molecule approaches. To this end, we emphasize in the course how single molecule approaches go hand in hand with traditional ensemble biophysical measurements. To reinforce the complementarity of approaches, we incorporated single-molecule approaches into homework projects such as write a one-page specific aims page for a NSF or NIH proposal on a molecular biology and/or cellular biology topic using single molecule approaches. In particular, having two course co-directors with complementary backgrounds in structural biology and single molecule approaches has served this purpose well.Some lectures are also given by faculty other than course directors, for example on specialized approaches that are not as widely used in biophysics yet, such as Teraherz spectroscopy. More important than the lectures by course directors and other faculty, student lectures are a major component of the course. Student overview lectures cover topics that exemplify the application of model systems in which a large number of different biophysical techniques have been applied to address biological functions. To assist students in formulating and addressing biological questions, we incorporate another major component, grant proposal writing, reviewing and addressing critiques. In addition to course directors, local faculty experts are used to assist students with tasks related to presentations and grant proposals. Finally, some major techniques are presented in hands-on demonstrations that result in generation of real data that is analyzed as part of the class. The analysis of the data acquired in class is given as homework. Additional homework includes reading of publications related to the lectures given by the students and the work related to the grant proposal materials. A grading scheme outlining the weight of the different components is provided in Figure 2B.

## Graduate student presentations

**Figure 3:**
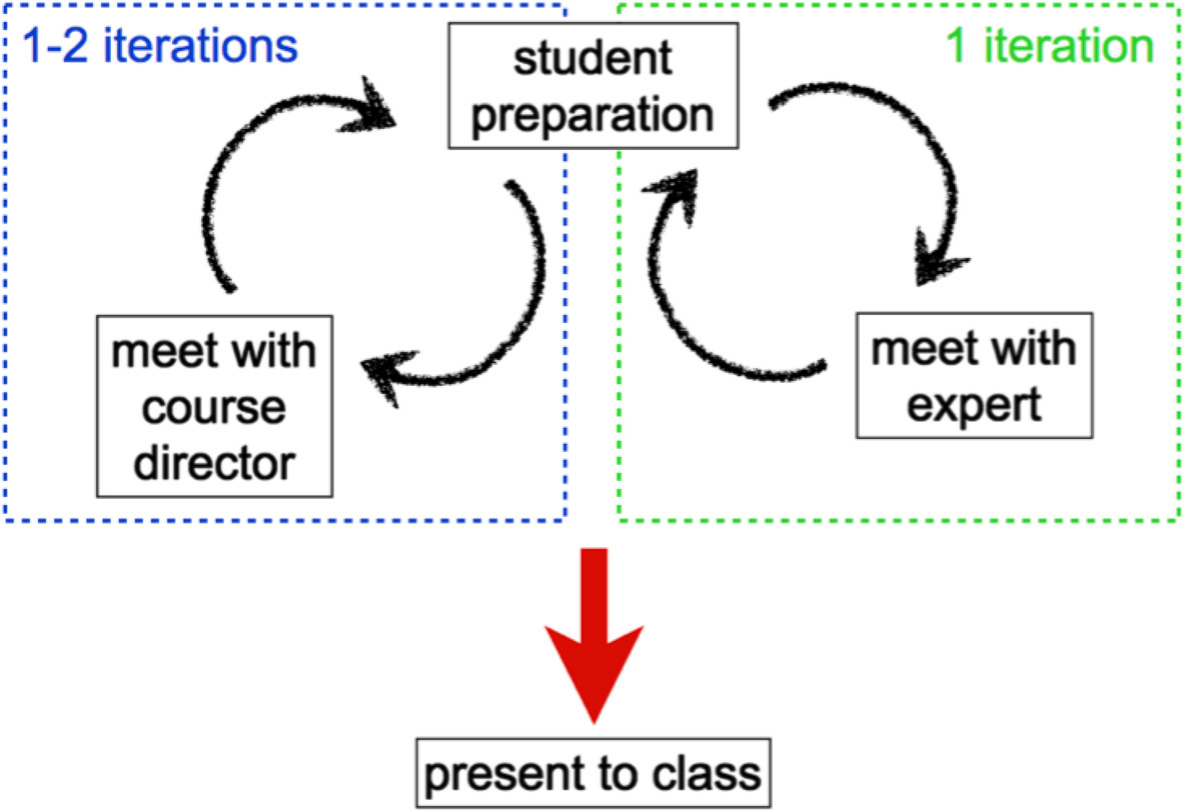
**Schematic outlining the preparation of student presentations with assistance from course directors and local experts.** The figure illustrates the procedure for the student presenter. Other students in the class need to read publications related to the presentation and prepare questions in advance (see text for details).

While many graduate level courses require student presentations about current literature, our approach is to ask the students to identify relevant literature beyond a single publication and synthesize information to be presented in class. Each student presents one class period. For each of the student lectures, the course instructors provide a short list of primary and/or review literature. The student that is to present needs to expand this list and synthesize the expanded material to fit into a 90-minute interval. There are several quality control levels incorporated (Figure 3). A course director reviews the first draft of the lecture, and 1–2 iterations are expected to finalize the lecture. To ensure that the student learns from the presentation preparation experience, we have each student presentation go through several practice iterations under individual faculty supervision before the actual presentation. Additionally, we seek the participation of local faculty experts. In practice, often students are presenting some of the work of a local expert, in context with the work from other scientists. First, the course directors work with the students to achieve a basic level of quality in terms of the presentation style and overall content, such that the external expert faculty can focus on the more detailed content in their area of expertise. Thus, the course directors oversee each student presentation before we allow the student to meet with the local expert. In the class in which the student presents, the local expert is invited to attend and asked to field questions that the student presenter cannot answer. Typically, these student presentations run for an hour and a half, including questions from the audience. For most students this is the longest presentation they have given, thus far, in their career.

## Local expert grant proposal

Most graduate programs incorporate a comprehensive exam, which typically involves writing a proposal. The MBSB program is no exception. At the end of the second year of study, the MBSB graduate students prepare a de novo 15 page NIH-style grant proposal based on one of three sets of one-page Specific Aims pages selected by a comprehensive exam committee. In addition to preparing the proposal, each student must defend the proposal in an oral examination with three MBSB faculty. Most students, including first year MBSB students, are typically unfamiliar with the process of writing a grant proposal with a one page specific Aims, introduction, rationale for each aim, description of the methods for each aim, discussion of the data expected to be obtained, discussion of pitfalls, timeline of experiments, and overall conclusions. We have therefore incorporated a professional grant proposal review component in the class. Students get to study real faculty proposals, particularly faculty proposals that have failed the first round of submission to NIH or NSF and then have succeeded in a subsequent resubmission. We solicit such proposals from colleagues (or use our own). A useful resource is the NIH website. A list of local successful A1 (second submissions to NIH) submissions at a university can be found using a query through NIH Reporter ( http://projectreporter.nih.gov/reporter.cfm). Upon identification of a successful A1 application, there is the need to sensitively approach the successful faculty to see if he or she would be willing to share their original A0 application, the successful A1 resubmission, and the two sets of peer review by the study section. Provided with these materials, we release them consecutively and chronologically to the students so that they may first write a critique of the original grant proposal before they see the report from the first study section reviews. At this point, the students then write a one to three page response to the original reviews as well as propose changes to the original proposal to satisfy the criticisms from the first review. Then the students are allowed to read the revised proposal and the subsequent reviews.

Classes are organized with one student presenting the original proposal with the local expert attending and with the other students having previously prepared questions. A subsequent class then includes a student presenting the revised proposal and the responses to the reviews, again with the local expert in attendance. Each student presentation goes through the same iterations used for all student presentations wherein the student meets with a class director one or more times before being allowed to meet with the local expert. In this way, students are exposed to the thought processes of faculty and how they respond to the criticism of expert reviewers; studying an existing successful grant proposal without the observation of the criticism, revisions, and responses to criticisms does not allow for as much development of the students.

An alternative to studying the development of grant proposal from failure to success is to invite a faculty member to introduce and discuss a subject (again having up to three publications provided to the students for them to prepare questions prior to the class), and afterwards requiring each student to synthesize three aims for a Specific Aims page. In this case, the best aims can be collated into an overall Specific Aims, and each student can diagram an experiment to best present that aim. Finally, the best experiments can be collated into the first version of a grant proposal.

Grant proposal sessions by students are typically run with local experts in attendance who then participate in a lively discussion on their proposals.

## Demo experiments

Faculty in the MBSB program cover a wide range of biophysical techniques, with some faculty using the same technique applied to different questions. For example, there are numerous faculty that use NMR spectroscopy as a major tool. We found that students often choose mentors based on techniques rather than topics. To help students make better choices and give them independence of gaining hands-on experience only in rotations, we incorporated a significant hands-on experience component into the class. Thus, we give several demos serving as introductions to the use of various instruments and major techniques. This allows for a novel strategy in interdisciplinary collaboration. Students trained in the basics of techniques with some hands-on experience are less hindered in seeking collaborations with faculty members in the future, even if they choose not to pursue a particular technique as their main technique used during their PhD. Collaboration between labs is encouraged in the MBSB program, and such knowledge should assist each student as they work on their thesis project as they may wish to collaborate or use the resources of various laboratories to finish their individual projects. Demos developed for the class include cryo-electron microscopy, solution nuclear magnetic resonance (NMR), solid state NMR, atomic force microscopy (AFM), single pair fluorescence resonance energy transfer (spFRET) and protein induced fluorescence enhancement (PIFE), protein crystallization and structure determination, and molecular modeling and other computational methods. Protocols used are based on those developed by the faculty presenting them, e.g., AFM protocols are described in [9].

The choice of experiments in these laboratory demos was carried out with a keen view on representation of state of the art techniques. For example, single molecule approaches such as AFM and single pair FRET and also cryo-EM are demonstrated alongside with ensemble-based techniques.

## Engaging students in discussions

Students who are not presenting are expected to be familiar with pre-selected papers on the topic that will be presented. In particular, the other students in the class are required to read one to three primary literature publications about the topic being presented and submit three thoughtful questions prior to class. Prior preparation of the list of questions is mandatory. The questions are the basis for the discussion of the material by the entire group of class attendants of that day (other students, course instructors, experts). The questions are graded to provide students with feedback. Good questions are those that seek to elucidate how the research can help describe mechanisms of molecular and cellular biology. Questions with less value are those that are purely technical, or obvious questions that have either already been answered in the literature or are not feasibly addressed. Additionally, the other students are graded on their participation in the class and encouraged to ask questions throughout the student lecture. The student lecture should not only give a solid background to the field but also analyze what are the unknown questions in the field. The addition of the local expert raises the level of each student presentation and benefits everyone.

## Teaching assistants

In some cases, senior MBSB graduate students have taught some of the demo experiments. They have also served as teaching assistants and assisted with evaluating student homeworks and questions, thus, providing a more complete experience. Student homeworks are of two types, analysis of data collected during wet-lab demo experiments and preparation of the student lectures, summaries and questions that serve as lecture notes. Students are expected to spend a large amount of time preparing the lectures thoroughly with close feedback loops to course directors and experts. Often, teaching assistants are a valuable resource to offer office hours at the computer cluster to help students in the analysis of data, or as a resource to provide feedback on the initial stages of lecture preparation.

## Complementary data and literature club

Because the class trains students to ask questions and critically analyze scientific data and literature, the skills learned in class can be reinforced when students attend lectures. Here, the Data and Literature Club provides a useful complementary addition to the MBSB curriculum. This club occurs one hour before the formal MBSB weekly seminar. One student presents 30 minutes on current data obtained in the laboratory, and the second student presents 30 minutes on a publication written by the MBSB seminar speaker of that day. Typically that student speaker contacts the seminar speaker ahead of time to get a suggested publication for presentation. For MBSB seminar speakers from outside Pittsburgh, the students share lunch with the speaker directly after seminar without other MBSB faculty present. The informal character of the lunch facilitates a more casual exchange of information, and external speakers have commended the high level of scientific discussion the students engage in with them. This routine benefits the MBSB graduate students in that they are exposed extensively to the field of study of the MBSB seminar speaker and provided with an opportunity to interact with faculty on a deeper level, directly applying the skills learned in the MB II course.

## Where are past PhD graduates of the MBSB program now?

Past MBSB PhD graduates have joined postdoctoral fellowships at the Cleveland Clinic, Duke University, Groningen University (The Netherlands), Harvard University, Ohio State University, Rockefeller University, Rush University Medical Center, and UCSF. One is performing his medical residency at the University of Pittsburgh. One is not yet employed. One is attending the Indian Institute of Management in India. One is an Instructor of Biology at Carlow College here in Pittsburgh.

## Feedback

We performed a survey on current and past graduate students who have taken the MBSB 2 class. 21 of 36 students responded anonymously (Additional file 1: Supplementary Information). In response to the question, "All in all – did you find this course useful?", 15 rated the class as "very useful", 5 rated the class as "somewhat useful", and one rated the class as "neutral, neither useful nor harmful". In response to the question, "What do you remember most vividly from the MBSB 2 course?", one student wrote, "One of the weekly assignments for the class was to read an article and submit three questions for each paper. The thing I most vividly remember from the class is the detailed feedback I received on my questions. By the end of the class, my grades on the questions had improved significantly due to the helpful commentary. I feel like this exercise in critical thinking really helped me as a scientist.

"I also remember the significant amount of time we spent reading, writing, and critiquing proposals. After the class I wrote (and received) a grant for a fellowship, and I know that what I learned in class benefited my proposal." For other responses to specific questions, please see the Additional file 1.

## Conclusions

We have briefly described an approach to teaching molecular biophysics and structural biology that requires a large involvement of the students in the class and minimizes faculty didactic lectures. We have had 36 graduate students take this class over the past 7 years. We are training the next generation of molecular biophysicists to function in a real world academic environment. The class has evolved over these 7 years and continues to evolve at present. For example, one module this semester was to prepare this paper as a class project.

Our desire for this class has been to integrate an array of complementary techniques, encourage collaboration and interdisciplinary research and explicitly train students in important skills for a research career: grant writing, presentation and out-of-the-box thinking. All these aspects are incorporated into our course that appears to ‘just’ teach the students about intermolecular interactions and dynamics, but has a deeper underlying goal in training the future generation of interdisciplinary biophysicists.

## Abbreviations

AFM: Atomic force microscopy; MB: Molecular biophysics; MBSB: Molecular biophysics and structural biology; NMR: Nuclear magnetic resonance; PIFE: Protein induced fluorescence enhancement; spFRET: Single pair fluorescence resonance energy transfer.

## Competing interests

The authors declare that they have no competing interests.

## Supplementary Material

Additional file 1 Supplementary information. Click here for file
