# Supplementary material for: Early integration of the individual student in academic activities: a novel classroom concept for graduate education in molecular biophysics and structural biology
Source: BMC Biophys. 2014 Aug 5;7:6. doi: 10.1186/2046-1682-7-6 (PMC4134111; doi:10.1186/2046-1682-7-6)
Supplement: Additional file 1 — Supplementary information. [file 2046-1682-7-6-S1.docx]

Supplementary Information

Early integration of the individual student in academic activities: a novel classroom concept for graduate education in molecular biophysics and structural biology

Sanford H. Leuba^*^, Sean M. Carney, Elizabeth M. Dahlburg, Rebecca J. Eells, Harshad Ghodke, Naveena Yanamala, Grant Schauer and Judith Klein-Seetharaman^*^

Graduate Program in Molecular Biophysics and Structural Biology at the University of Pittsburgh and Carnegie Mellon University, Pittsburgh, PA 15260, USA

*To whom correspondence should be addressed:

Sanford H. Leuba, Department of Cell Biology, University of Pittsburgh School of Medicine, UPCI, Hillman Cancer Center, 5117 Centre Avenue, Pittsburgh, PA 15213, USA, Tel:+1-412-623-7788; Email: [leuba@pitt.edu](mailto:leuba@pitt.edu) and Judith Klein-Seetharaman, formerly Department of Structural Biology, University of Pittsburgh, now Professor of Biomedicine and Systems Biology, Division of Metabolic and Vascular Health, Warwick Medical School, University of Warwick, Coventry, CV4 7AL, UK; Tel:+442476573806; Email: j.klein-seetharaman@warwick.ac.uk.

Sean M. Carney, scarney710@gmail.com

Elizabeth M. Dahlburg, bethdahlburg@gmail.com

Rebecca J. Eells, rje14@pitt.edu

Harshad Ghodke, hghodke@gmail.com

Naveena Yanamala, naveena.somisetty@gmail.com

Grant Schauer, grantschauer@gmail.com

**Results from MBSB 2 Survey**

We prepared an anonymous survey to ask students who have taken the MBSB 2 class. 21 of 36 students responded. 15 students rated the class overall as “very useful”, 5 students rated the class overall as “somewhat useful”, and 1 student rated the class overall as “neutral, neither useful or harmful”.

**1. What do you remember most vividly from the MBSB2 class?**

**Written responses from 15 students who rated the class as “very useful”.**

One of the weekly assignments for the class was to read an article and submit three questions for each paper. The thing I most vividly remember from the class is the detailed feedback I received on my questions. By the end of the class, my grades on the questions had improved significantly due to the helpful commentary. I feel like this exercise in critical thinking really helped me as a scientist. I also remember the significant amount of time we spent reading, writing, and critiquing proposals. After the class I wrote (and received) a grant for a fellowship, and I know that what I learned in class benefited my proposal.

How to effectively communicate your science and research? Biophysical techniques with and hands on problem solving approach. Teaching us the pros, cons and effective ways of grant writing and evaluation, indirectly teaching us how to formulate a research idea for grant applications in future.

Hands on experience with techniques and critiques and reviews of real grants.

Feedback on journal club questions and in depth research talks presented by students as course project.

Preparing a lecture on voltage-gated potassium channels to educate the class on topic and the current hypotheses in the field.

The quality of the lectures delivered by the experts of the field was top notch and the discussion on these topics were almost always stimulating. The topic reviews we have worked on helped us put the various techniques in context of real research problem.

The collaborative learning experience. It was very helpful to discuss, and figure out, questions as a group. I enjoyed not having to drudge through discussions until we came to “the” answer, but instead worked together to understand “an” answer.

Interactive nature of the class; focus on student driven presentations.

The most memorable aspect was delving into a real grant application. Reading the grant and responding to actual reviewer critiques was incredibly useful. Presenting this in front of the grant’s author as well as my colleagues with a focus on public speaking skills was also a memorable exercise.

The grant reviews and the live demos of X-ray & NMR.

The introduction to a variety of different grants, ranging from NIH R01’s to NSF career awards, etc.

Differences in single molecule versus bulk biophysical experiments.

All the hands on experience, along with visiting different labs. Doing protein crystallography from crystal formation through side chain correction. Monte with Gordon Rule. Phases with Bill Furey.

Student presentations and asking three questions for every paper. And the best part; learning the practical aspects of the various biophysical methods we were introduced to in MBSB 1.

We had to present a paper and an in house guest expert on the subject of the paper was invited to participate in discussions. The speaker was given a change to interact with the expert while preparing for the seminar. The second thing that I remember is when we had to come up with novel ideas for a grant and present in the class for discussion.

**Written responses from 5 students who rated the class as “somewhat useful”.**

Evaluating the R01 proposals and preparation for the student lectures.

The stress of putting together perfect presentations.

Reading a lot of papers in a short amount of time (which was good preparation).

It was very challenging as students have to present and there was homework every week. It was a good experience in speaking and analytical thinking skills.

**Written response from 1 student who rated the class as “neutral, neither useful or harmful”.**

The class was disorganized and did not successfully accomplish the goals set forth at the beginning of the semester. Year to year, the course was inconsistent and was sometimes more successful than others.

**2. Do you think any decision about your career was influenced by this course? If yes, please describe.**

**Written responses from 15 students who rated the class as “very useful”.**

The class influenced my decision to receive training in experimental biophysics methods to complement by computational training.

Applying multidisciplinary approaches to address a particular problem in science.

The course expanded my understanding of possibilities and limitations of multiple experimental techniques, but this new understanding confirmed my career intentions not changed them.

In designing graduate level courses myself, I aim to incorporate the lecture styles of this course.

I don’t think I have based any decisions directly off of the course. However, the class did provide a valuable experience into the writing and reviewing process for research funding grants; a very valuable experience to me.

The course gave me more confidence to continue pursuing a career in academic research. By exposing me to grantsmanship (easily one of the most important aspects of professorship) in a realistic way, I was able to see that I was capable of handling similar tasks.

This course and the related MBSB courses deepened my awareness of and love for molecular biophysics. I am still uncertain with what the exact future will hold for me.

I think it was during this MBSB II class that I realized that I would not want to be in a academia after getting my Ph.D.

**Written responses from 5 students who rated the class as “somewhat useful”.**

It was very interesting to learn about the funding process as well as the challenges in writing a proposal. I always assumed that the process was very challenging, but learning about how and why these processes are challenging was a valuable experience.

**Written response from 1 student who rated the class as “neutral, neither useful or harmful”.**

None of my career decisions were influenced by this course.

**3. Did you find this course in any way unusual. If yes please describe what elements of this course are different from other courses you have taken or heard about.**

**Written responses from 15 students who rated the class as “very useful”.**

I think this course was unusual in the amount of time and effort that wad dedicated to making us better scientists. To me, learning biophysical methods seemed like a side-benefit of the class, where the real focus was on scientific communication and thought.

This course has taught us cutting edge methods with hands on experience. Teaching using and working by example model is always useful to grasp concepts with ease.

The grant critique and review aspect of the course was refreshing. I had not heard about any other class that did that with real PI’s grants.

There was a lot of feedback on our exams, presentations, and journal club questions. I really liked this and think more courses should follow this example.

The hands-on demos and data analysis assignments were unusual, and ended up being useful in my own work down the road as I now have to analyze ARM data using the same or similar approach to what was assigned in the class.

The course promoted scientific thinking and discussion skills. We were required to prepare questions of the pre-assigned readings. This thinking process forced us and helped us to find the way of how to ask good scientific questions.

The practical aspects of the course content. Unlike many typical university coursed that focus on memorization and regurgitation of facts, this course focused on critical thinking and asking “how” and “why” questions as opposed to just “what”.

Interactive nature of the class; focus on student driven presentations; Focus on critical thinking.

I felt that this course had an earnest goal of training the students as tenure-track professors in academia. Whereas other graduate courses focus on knowledge base and/or skill sets, the focus of this course definitely made it unique.

More interactive, more hands-on experience and the experience with writing grants.

The course did not seem to fit the mold of a typical graduate course. It seemed to feel very much like a grant writing worship. It was unique in this perspective since it introduced us to grant writing and reviewing. Few courses offer this.

Assessing student knowledge on the first day of class by a pop quiz and adjusting the course curriculum accordingly, modular course structure and promoting and actively engaging students in each lecture.

This course, along with the other MBSB course was challenging and engaging in ways I have not experienced otherwise. The pantheon of amazing materials from first-rage minds forms a broad overview of life with enough details to competently explore more.

It was different in that we got to see the practical aspects of so many different biophysical methodologies. Usually a class focuses on one or two, so the breadth of the course was something I liked. I thought the mandatory formation of three questions for every reading was weird but it did teach me to ask questions a I read papers. In the future though I would limit it to one question so I can focus on coming up with one great question rather than three good questions.

This course was more interactive than other courses.

**Written responses from 5 students who rated the class as “somewhat useful”.**

Between the coursework involving the R01’s and the demonstrations, the class seemed to focus on delivering direct experience as opposed to simply following a textbook.

It was more hands-on with the demos, which is a positive thing.

I liked the way in which all the presentations were student-driven.

The course was unique as it had no preplanned syllabus. We had new lecturers for every module and students got practical experience in the techniques along with critiquing grants and papers.

It was not a typical graduate course in terms of the type of work we did and how we were evaluated. It resembled more of a workshop/seminar than a typical graduate course.

**Written response from 1 student who rated the class as “neutral, neither useful or harmful”.**

This course encouraged significantly more student involvement than others, however that often allowed students to take advantage and steer the course off track. This approach would work best if students were all self-motivated, which is not always (or often ) the case.

| “Yes” or “kind of yes” or “neutral, neither yes or no” or “no” responses from students to specific questions: | From the students who rated the class very useful | From the students who rated the class somewhat useful | From the students who rated the class neutral, neither useful or harmful |
| --- | --- | --- | --- |
| Did the course  enhance your scientific discussion skills? | 13 yes  2 kind of yes | 5 kind of yes | 1 kind of yes |
| Did the course help you design experiments outside the course? | 7 yes  4 kind of yes  3 neutral, neither yes or no  1 no | 3 kind of yes  1 neutral, neither yes or no  1 no | 1 no |
| Did you have more confidence discussing science in public as a result of this course. | 10 yes  5 kind of yes | 2 yes  2 kind of yes  1 neutral, neither yes or no | 1 kind of yes |
